# The role of goblet cells in Crohn’ s disease

**DOI:** 10.1186/s13578-024-01220-w

**Published:** 2024-04-01

**Authors:** Zichen Wang, Jun Shen

**Affiliations:** 1 Division of Gastroenterology and Hepatology, Baoshan Branch, Renji Hospital, School of Medicine, Shanghai Jiao Tong University, Shanghai, China; 2 Division of Gastroenterology and Hepatology, Key Laboratory of Gastroenterology and Hepatology, Inflammatory Bowel Disease Research Center, Renji Hospital, School of Medicine, Ministry of Health, Shanghai Jiao Tong University, Shanghai Institute of Digestive Disease, No.160 PuJian Road, Shanghai, 200127 China

**Keywords:** Crohn’s disease, Intestinal epithelial cells, Goblet cells, Inflammatory bowel disease

## Abstract

The prevalence of Crohn’s disease (CD), a subtype of inflammatory bowel disease (IBD), is increasing worldwide. The pathogenesis of CD is hypothesized to be related to environmental, genetic, immunological, and bacterial factors. Current studies have indicated that intestinal epithelial cells, including columnar, Paneth, M, tuft, and goblet cells dysfunctions, are strongly associated with these pathogenic factors. In particular, goblet cells dysfunctions have been shown to be related to CD pathogenesis by direct or indirect ways, according to the emerging studies. The mucus barrier was established with the help of mucins secreted by goblet cells. Not only do the mucins mediate the mucus barrier permeability and bacterium selection, but also, they are closely linked with the endothelial reticulum stress during the synthesis process. Goblet cells also play a vital role in immune response. It was indicated that goblet cells take part in the antigen presentation and cytokines secretion process. Disrupted goblet cells related immune process were widely discovered in CD patients. Meanwhile, dysbiosis of commensal and pathogenic microbiota can induce myriad immune responses through mucus and goblet cell-associated antigen passage. Microbiome dysbiosis lead to inflammatory reaction against pathogenic bacteria and abnormal tolerogenic response. All these three pathways, including the loss of mucus barrier function, abnormal immune reaction, and microbiome dysbiosis, may have independent or cooperative effect on the CD pathogenesis. However, many of the specific mechanisms underlying these pathways remain unclear. Based on the current understandings of goblet cell’s role in CD pathogenesis, substances including butyrate, PPARγagonist, Farnesoid X receptor agonist, nuclear factor-Kappa B, nitrate, cytokines mediators, dietary and nutrient therapies were all found to have potential therapeutic effects on CD by regulating the goblet cells mediated pathways. Several monoclonal antibodies already in use for the treatment of CD in the clinical settings were also found to have some goblet cells related therapeutic targets. In this review, we introduce the disease-related functions of goblet cells, their relationship with CD, their possible mechanisms, and current CD treatments targeting goblet cells.

## Abbreviations

AGR2 anterior gradient protein 2;

AIEC adherent-invasive *Escherichia coli;*

AKT protein kinase B;

CD Crohn’s disease;

COX2 cyclooxygenase 2;

DSS dextran sulfate sodium;

EHEC enterohemorrhagic *Escherichia coli;*

ER endoplasmic reticulum;

ERK extracellular signal-regulated kinase;

Fxr farnesoid X receptor;

GAP goblet cell-associated antigen passage;

IBD inflammatory bowel disease;

IEC intestinal epithelial cell;

IFN interferon;

IL interleukin;

IL-13Rα2 IL-13 receptor alpha 2;

ITF intestinal trefoil factor;

LPS lipopolysaccharide;

MUC2 mucin 2;

NF-κB nuclear factor-kappa B;

NLRP6 NOD-like receptor family pyrin domain containing 6;

NO nitric oxide;

NOD2 nucleotide-binding oligomerization domain-containing protein 2;

PHB1 prohibitin 1;

PI3K phosphoinositide 3-kinase;

PKC protein kinase C;

RELEM-β resistin-like molecule beta;

ROS reactive oxygen species;

STAT signal transducer and activator of transcription;

TFF3 trefoil factor 3;

TGF-β transforming growth factor-beta;

TLR Toll-like receptor;

TNBS trinitrobenzene sulfonic acid;

TNF-α tumor necrosis factor;

TRIM21 tripartite motif containing-21;

## Background

Crohn’s disease (CD) is a chronic inflammatory disorder affecting any part of the gastrointestinal tract, with common symptoms including chronic diarrhea, abdominal pain and weight loss [1]. CD may also lead to complications outside of the gastrointestinal tract, including anemia, rash, diarrhea and fatigue. For CD patients, colorectal cancer also poses a potential hazard [2]. CD is diagnosed after a comprehensive evaluation, which is based on the laboratory test, endoscopy and imaging studies [3]. Laboratory test should involve basic assessment of inflammation, anemia, malnutrition and dehydration. Stool test is required for active CD patients, which usually involve pathogen test and Clostridium difficile test. Gut inflammation markers such as fecal calprotectin is also recommended. Ileocolonoscopy should be done with biopsies for the suspected CD. During the endoscopy examination, the location, severity of the lesion and mucosal healing status should be recorded. Computed tomography enterography or magnetic resonance enterography should be performed for imaging studies. After taking a thorough consideration of all the test results, the diagnosis of the CD can be made [3]. CD prevalence and incidence rate are increasing worldwide with a prevalence in developed countries of 1 in 200 individuals [4, 5]. However, its prevalence in new industrial countries has also recently increased [6]. CD pathogenesis is thought to be related to genetically susceptible individuals developing aggressive acquired immune responses to commensal enteric bacteria under specific environmental factors [7]. Hence, genetic, bacterial, mucus barrier, and immune factors all contribute to CD etiology. In particular, the normal functions of the epithelial barrier and mucosal immune defense become disrupted [1].

In the gut, the intestinal epithelial cells interact directly with the gut lumen and comprises enterocytes, Paneth cells, goblet cells, tuft cells, microfold (M) cells and enteroendocrine cells [8]. Given that these cells play vital roles in maintaining homeostasis, some may contribute to CD progression. Indeed, Paneth cells are related to several CD susceptible genes [9], while damage to the tight junctions between epithelial cells facilitates the translocation of bacteria from intestinal lumen [10]. Additionally, goblet cells and intestinal epithelial cells that secret mucus may be associated with CD. Previous research revealed that goblet cells also exhibit functions other than mucus secretion, which might impact CD etiology factors differently [11]. Therefore, goblet cells might be associated with CD disease progression, which was not paid much attention to in previous research.

In this review, we discuss the relationship between goblet cells and CD. In particular, we review the functions of goblet cells that have been discovered in previous research. We discuss and analyze how the goblet cells dysfunction may aggravate the etiology of CD in pathogenic situations. Based on these mechanisms, we introduced the current and potential pharmacological treatments related to goblet cells.

## Physiologic function of goblet cells

**Fig. 1:**
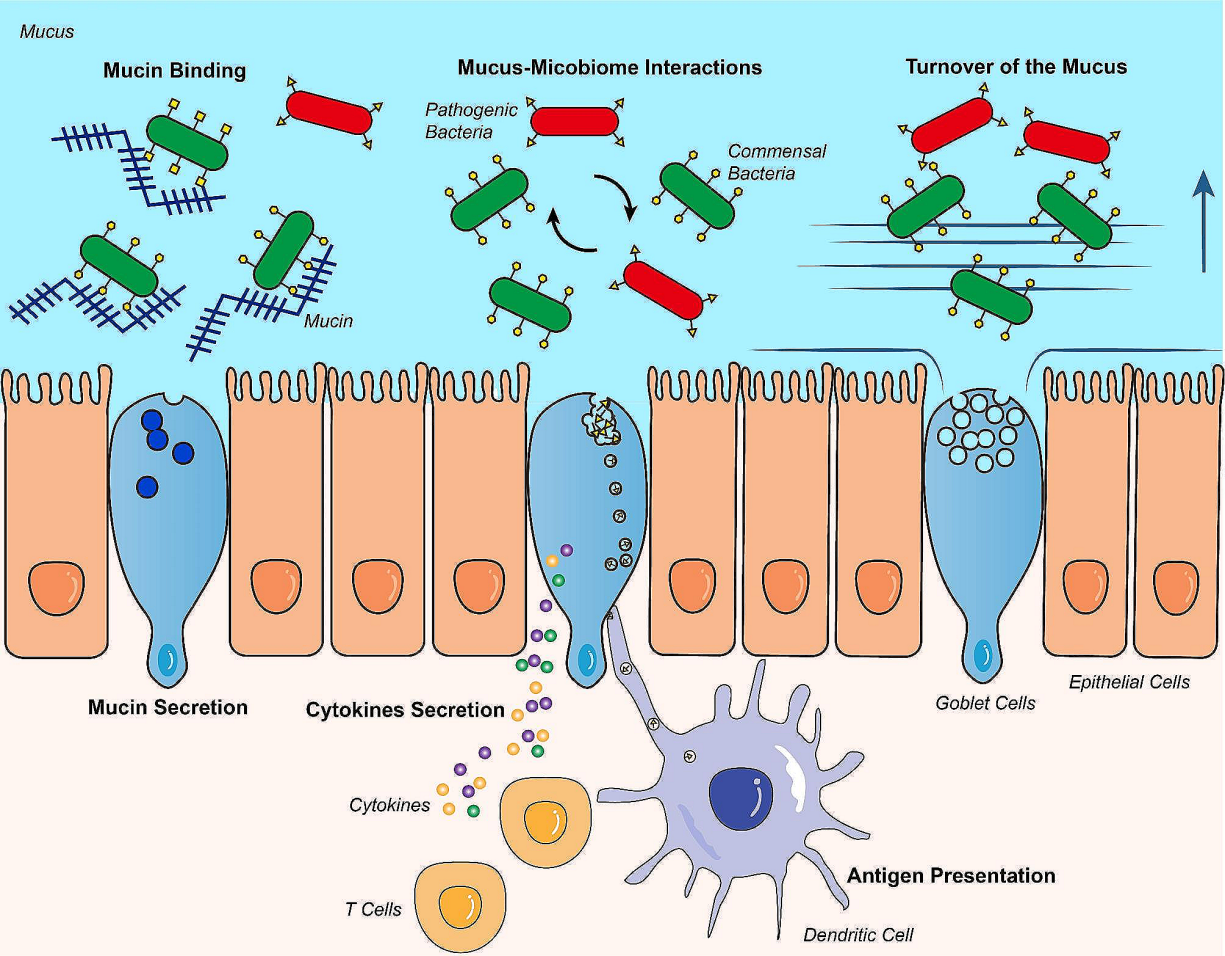
Functions of goblet cells. Goblet cells have several functions which are directly or indirectly related to Crohn’s disease: ① Mucus secretion: goblet cells secret and renew the mucus layer at a steady turnover rate. This secretion process also pushes most pathogenic bacteria away from approaching the enterocytes. ② Mucin secretion: goblet cells secret mucins which have binding sites for bacteria. Mucins play a vital role in anti-pathogenic bacteria invasion. ③ Mucus-Microbiome Interactions: Bacteria take substance in the mucus as energy source for their growth and affect the mucus secretion. Mucus determines the microbiome constitution in gut. ④ Interaction with the immune system: goblet cells are involved in antigen presentation process, and they also secret cytokines, interfering the immune system

Goblet cells have various functions. In this review, we mainly focus on the functions that might be associated with the pathophysiology of CD, which include mucus and mucin secretion, antigen presentation and mucus barrier maintenance (Fig. 1).

Previous research primarily focused on various intestinal epithelial cells, excluding goblet cells. For instance, paneth cells have been associated with multiple genes linked to CD susceptibility [9], and damage to the tight junctions between epithelial cells can facilitate the migration of bacteria from the intestinal lumen [10]. While the mucus secretion function of goblet cells has long been recognized, their connection to CD pathogenesis was not given much attention in the past. However, recent studies increasingly reveal that goblet cells possess additional functions beyond mucus secretion. These functions may impact different factors contributing to the development of CD [12]. Consequently, the relationship between goblet cells and the progression of CD might be significantly stronger than previously believed. In the following sections, we will explore several functions of goblet cells related to the pathogenesis of CD, which focus on its influence on the mucus barrier, immune system and gut microbiota.

### Influence on the mucus barrier

Mucus protects the gut from gastric acids and digestive enzymes [13]. Most importantly, mucus builds up a barrier against bacteria and their by-products. For the structure of the mucus barrier, it differs between the small and large intestines [14], causing the mucus in the small intestine is to be thin, while that in the colon is thick and double-layered [15]. The distribution of goblet cells and mucus differs between the small intestine and colon with their abundance gradually increasing from the small intestine to the colon. The composition of mucin also varies between the small intestines and colons. This difference is primarily attributed to the structural dissimilarity in the mucus layer. In the small intestine, a single mucus layer is predominantly formed by MUC2 [16]. Conversely, in the colons, the upper layer of mucin composition is similar to that of the small intestine, mainly consisting of MUC2 [16]. Porchet et al. noted that MUC5B is also weakly expressed in the colon [17]. However, the inner layer of mucin composition differs, primarily comprising various transmembrane mucins such as MUC3A/B [16], MUC4 [17], MUC12, MUC13 [18], MUC15 [19], MUC17 [20], and MUC20 [21]. These transmembrane mucins form a dense glycocalyx layer, creating a barrier that separates the colon epithelial cells from the upper loose and permeable mucus layer. The upper layer of mucus in the colon is colonized with bacteria, while the inner layer is impenetrable to microorganisms. The thin layer in the small intestine may have a protective function while also facilitating the absorption of nutrients.

The mucosal barrier is a superficial barrier that directly interacts with the environment that exerts physical and biochemical effects. Goblet cells maintain an intact mucus layer by synthesizing, secreting, and degrading it at a steady turnover rate [22]. A Previous study observed GalNAz protein-labeled MUC2 via fluorescence microscopy and found its synthesis to be fastest at the luminal surface (3 h before secretion) and slowest at the crypt epithelium (6 to 8 h before secretion) [23]. This variance in synthesis speed leads to the migration of the microbiome from the intestinal epithelium to the lumen. Meanwhile, another study investigated the permeability of mucus to microparticles by evaluating the diffusion of negatively charged latex beads into the swine intestine [24]. They found that the mucus structure was arranged three-dimensionally with small poles and that the permeability depended on the electrostatic charge. In this way, the stability of the gut microenvironment is maintained by the microstructure, electrostatic charge, and renewal of mucus by goblet cells.

Goblet cells can also biochemically affect mucus function. Mucins, including MUC, trefoil factor 3 (TFF3), etc., are the main components of mucus [25]. During the stimulated secretion process, luminal antigens, including lipopolysaccharide (LPS) and P3CSK4, are endocytosed by sentinel goblet cells, activating the downstream signaling pathway. Subsequently, the autophagosome generates reactive oxygen species (ROS), leading to the activation of NOD-like receptor family pyrin domain containing 6 (NLRP6)-mediated caspase 1 and caspase 11 regulation of Ca^2+^ levels and MUC2 exocytosis [26, 27]. This route mainly utilizes exocytosis for mucin secretion. Based on previous research, besides compound exocytosis, vesicle secretion is another main mechanism of mucin secretion by goblet cells [28]. Both of the compound exocytosis and vesicle secretion rely on vesicular transport. The process of mucin vesicle secretion in airway goblet cells is enhanced by certain components such as syntaxins, VAMP, SNAP, and Munc18 [29]. Gastrointestinal goblet cells may follow a similar regulatory method [28]. During exocytosis, the main mediators are intracellular Ca^2+^ levels and substances that can induce Ca^2+^ mobilization, such as histamine and acetylcholine [30]. When discussing mucin secretion during pathogen infection, it was found that the colon’s mucin vesicle secretion is triggered to prevent ischemia-induced pathogen invasion [31]. Moreover, vesicle-dependent mucin secretion regulator was also studied. It was indicated that unconjugated microtubule-associated-protein light-chain cannot be converted into its conjugated forms without the NLRP6 inflammasome, leading to impaired MUC secretion [32]. Since NLRP6-deficient mice exhibit suspended autophagy, autophagy may regulate vesicle-dependent mucin secretion.

### Influence on the immune system

Goblet cells have the ability to interact with the immune system directly, fulfilling crucial roles in processes such as antigen presentation and cytokine production. It is indicated that goblet cells are involved in the endocytosis of antigens in the gut [33]. For the specific route, it has been established that goblet cells can transport antigens to CD103^+^ dendritic cells, which are activated by muscarinic acetylcholine receptors binding to acetylcholine and IL-13 [34]. Conversely, the epidermal growth factor receptor (EGFR) and Toll-like receptors (TLR) -2, -4, and − 5 impede the antigen- presenting process on goblet cells [34, 35].

Moreover, goblet cells also have a vital role in cytokine-mediated immune responses. In particular, the levels of IL-6, IL-7, IL-13, IL-15, IL-17, IL-18, IL-25 and C-C motif ligand 6 (CCL6) was elevated during goblet cell- associated antigen presentation, which may be related to the recruitment of T-cells and antigen presenting cells [35, 36]. Indeed, Th1 cells become activated following recognition of presented antigens and, subsequently, secret IL-1β and tumor necrosis factor (TNF) α stimulating goblet cells to secret MUC2. These processes are regulated by phosphoinositide 3-kinase (PI3K)/protein kinase B (AKT) and protein kinase C (PKC)-MER/extracellular signal-regulated kinase (ERK) signaling pathways [37–39].

### Interactions with intestinal microbiota

Mucus secreted by goblet cells bidirectionally interacts with the microbiota in the intestine. In one direction, Mucus provides gut bacteria with the nutrients and microbiome they need to survive, ensuring gut commensalism. Some specific bacterial species, such as *Bacteroides thetaiotaomicron*, can be influenced by goblet cells [40]. Moreover, the interaction between mucin and bacteria was also studied, which show the vital role mucin play in shaping the gut microbiome and influencing bacterial behavior in the intestinal environment. To be more specific, gut microorganisms adhere to glycan-rich sites on mucin, which mainly serve as a way for gut protection [41–43]. The adhesion is typically achieved by the pili or flagella structures of bacteria [44, 45], leading to a relatively fixed composition of the gut microbiome [46]. In addition to the well-known adhesion methods involving *flagella*, *fimbriae*, and *pili*, microbiota can also employ alternative adhesion mechanisms. A previous study revealed that mucus-binding proteins can assist in the adhesion process of *Lactobacillus reuteri* to mucin [47]. Certain microbiota, such as *E. coli*, produce adhesin proteins like FimH, which allow them to attach to the mucus layer [48]. Moreover, lectin-like mucus adhesins are another way microbiota can adhere to mucin. These lectin-like proteins specifically bind to glycoproteins present on the mucin surface [49]. Besides protecting intestine from invasion through adhesion, glycine-rich mucin could also be cleaved by microorganisms via glycosidases to generate energy [50]. In particular, various commensal bacteria, such as *Akkermansia muciniphila* [51], *Bacteroides fragilis* [52] and *Ruminococcus torques* [51], utilize cleaved mucins as an energy source. The commensal bacteria belonging to the *Verrucomicrobia phylum*, *Actinobacteria phylum*, *Bacteroidetes phylum*, and *Firmicutes* have been shown to possess the ability to degrade mucin. This degradation can occur through either an independent pathway or by interacting with other members of the microbiome. These commensal bacteria rely on the nutrients provided by mucin for their colonization [53]. However, certain pathogenic bacteria, including enterohemorrhagic *Escherichia coli* (EHEC) [54], also similarly obtain energy sources through this pathway. The commensal and pathogenic bacteria could compete for mucin as the energy source, leading to the poor colonization of the opponent. Thus, this competition could lead to the uncontrolled cleavage, exaggerated dysbiosis and mucin degradation finally. Previous studies have shown a close relationship between the composition of commensal bacteria, mucin production, and the pathogenesis of CD [55]. There was a significant reduction in the abundance of *Oscillospira* and *Akkermansia phylum* in CD. The reduction in both phyla could lead to more mucin cleaved by the pathogenic bacteria and is closely associated with mucin degradation. Furthermore, this reduction contributes to increased intestinal permeability [56]. Another type of bacteria involved in mucin degradation is sulfate-reducing bacteria, which produce hydrogen sulfide (H2S) during the process of sulfate reduction. H2S induces cytotoxicity in the intestinal epithelial cells (IECs) [57]. Consequently, the mucin-degrading process caused by bacteria ultimately leads to the pathogenesis of CD.

In another direction, gut bacteria also influence mucus secretion. Commensal bacteria occupy the adhesion sites of mucin and increase mucus secretion to avoid pathogenic bacteria invasion [58]. For instance, the commensal bacterium *Bacillus thetaiotaomicron* induces goblet cell differentiation and expression of mucus-associated genes [59]. Meanwhile, pathogenic bacteria can also have direct or indirect effects on the mucus secreted by goblet cells. For instance, TOXA secreted by *C. difficile* reduces mucin exocytosis by goblet cells, leading to a dysfunctional mucus layer [60]. While the *E. coli* strain LF82 degrades mucin through the protease VAT-AIEC, which is closely related to CD pathogenesis [61].

Moreover, microbes can also influence the growth and development of goblet cells. Previous animal experiments have shown that germ-free mice have fewer and smaller goblet cells [62], which dependents on the aryl hydrocarbon receptor relating to xenobiotic indole compounds [63]. Besides, the primary and secondary metabolites produced by commensal bacteria can also stimulate the differentiation of goblet cells and the secretion of mucus. Metabolites such as histamine, taurine, and spermine, generated by commensal bacteria, enhance mucus secretion through a pathway associated with the NLRP6 inflammasome [64]. Microbes also play a role in controlling mucin secretion to some extent. Previous research has reported that short-chain fatty acids produced by bacteria can stimulate goblet cells to secrete mucin [65]. The expression of the MUC2 gene can be upregulated by short-chain fatty acids derived from the microbiome, through histone acetylation or methylation of the MUC2 promoter and the AP-1 pathway [66]. Thus, abnormalities in the development and secretion of goblet cells caused by microbes may result in decreased mucin secretion, potentially contributing to CD development.

Surprisingly, specific bacteria can exploit the goblet cell-associated antigen passage (GAP) to penetrate the mucus barrier and traverse intestinal epithelial cells. Several studies have reported that *Listeria monocytogenes* [67–70], *S. typhimurium* [69, 71], *Shigella flexneri* [72] and *E. coli* [73] exhibit GAP-related bacterial translocation using goblet cells, which is initiated by the binding of bacteria to E-cadherin during mucus or MUC2 secretion.

## Goblet cell and Crohn’s disease

**Fig. 2:**
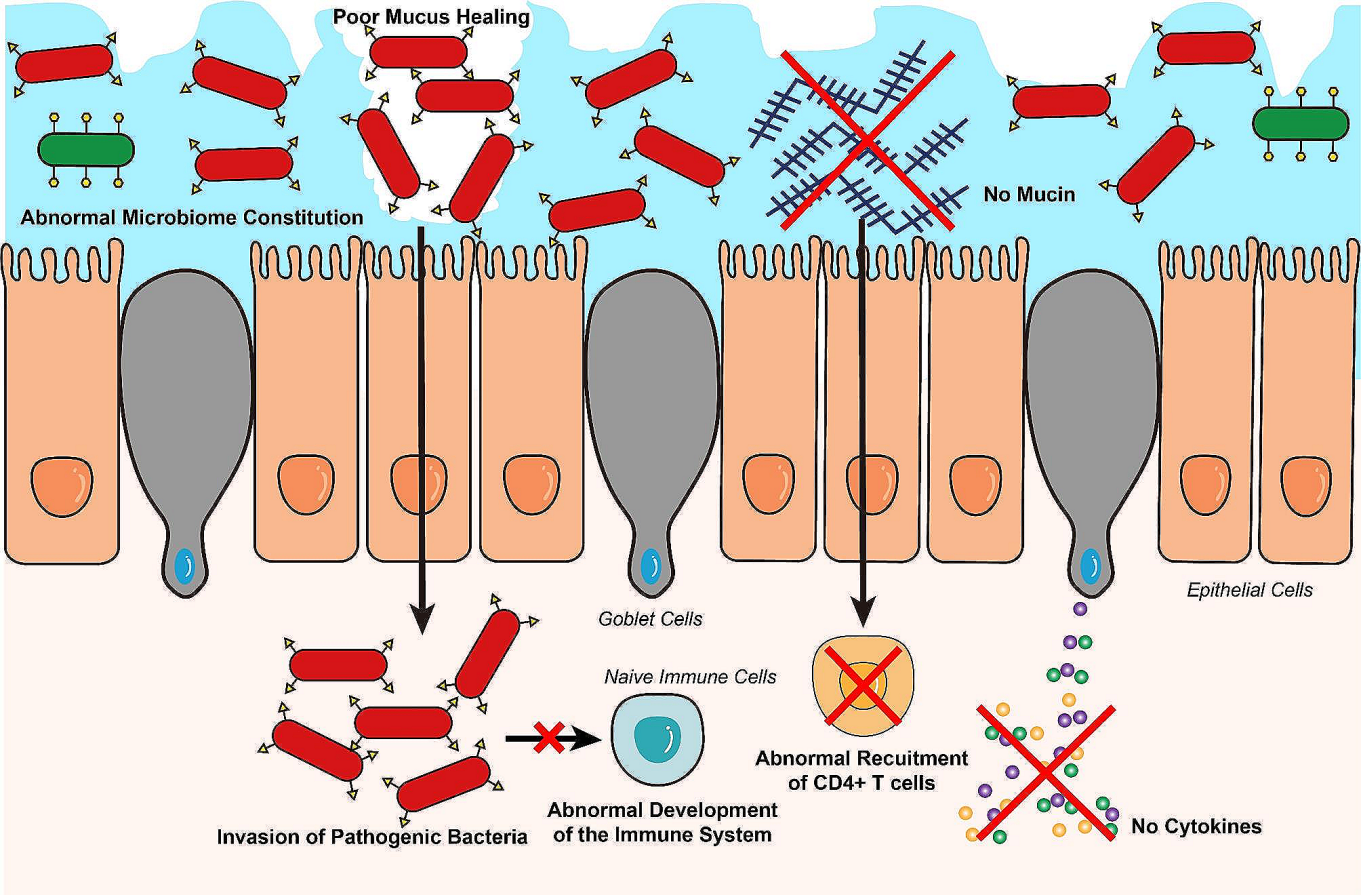
Dysfunctional goblet cells and Crohn’s disease. Dysfunction of goblet cells will cause a series of consequences which could lead to the pathogenesis of the Crohn’s disease. Dysfunctional goblet cells will cause poor mucus healing when the mucus secretion is abnormal. This will further lead to the pathogenic bacteria invasion into inner environment of enterocytes. The aftermath of the invasion is that inflammatory response was turned on and the normal development of the immune system was suspended. Mucins and cytokines secretion are also ceased. Recruitment of CD4^+^ cells stopped since mucins like RELEMβ has a regulatory effect on this process. All of these will lead to the abnormal immune reaction in the enterocytes and cause Crohn’s disease

The etiology of Crohn’s disease is associated with genetic, bacterial, mucus barrier, and immune factors. Compared to ulcerative colitis (UC), goblet cells’ role in animal CD models differs. In UC, there appears to be more severe and widespread goblet cells dysfunction, which is characterized by reduced mucin production that compromises the barrier function. As previously mentioned, goblet cells have various functions, which include influencing the immune system, interacting with the bacteria and interfering the mucus barrier etc. Thus, we can see a clear association between the goblet cells function and CD etiology. Abnormal functions of goblet cells are directly or indirectly related to pathogenic CD factors, which will be discussed respectively (Fig. 2). For the genetic factors for CD, the role of goblet cells is mainly the downstream effector of these susceptible gene. Thus, we give a quick glance here. Previous research indicated that some susceptible genes of CD may be related to goblet cell function. The CD specific loci are found in ATG16L1 and Nucleotide-binding oligomerization domain-containing protein 2 (NOD2) genes in Genome-wild association studies, which proves these two key genes are closely linked with the development of CD [74]. ATG16L1 is involved in the autophagy process, responsible for recycling cellular components and reducing endoplasmic reticulum (ER) stress. Defective ATG16L1 leads to abnormal mucus secretion and causes CD [75]. NOD2 is another gene that is linked to goblet cells dysfunction. Research by Maria Naama et al. has indicated that NOD2 may disrupt mucus secretion in goblet cells through the autophagy-related process as well [76]. NOD2 could also affect cytokine production of innate immune cells [76, 77], which may indirectly control goblet cells’ function.

Thus, the role of goblet cells is mainly the downstream effector of these susceptible gene. In the following sections, we are going to introduce the goblet cell’s possible role as an upstream effector, which is tightly linked with goblet cells’ physiology mentioned above. We will introduce goblet cells direct or indirect influence on the barrier function, immune response, autophagy and microbiome during CD pathogenesis.

### Abnormal goblet cell’s function may interfere the mucus barrier normal function, leading to CD pathogenesis

Mucus secreted by goblet cells is essential for maintaining microbiome stability. In a previous study, rectal biopsies of 59 patients with CD were analyzed, and mucus thickness and goblet cell density were measured [78]. Results show that the mucosal layer is thinner in active-stage patients. Moreover, the mucus barrier is typically not intact in CD, allowing the translocation of pathogenic bacteria, resulting in the activation of pro-inflammatory immune responses and CD development [79]. This has also been demonstrated using animal models [80]. Meanwhile, Wyatt et al. conducted a study on 72 patients with quiescent CD who underwent a lactulose-mannitol test to measure intestinal permeability [81]. The results showed that increased intestinal permeability is strongly correlated with CD relapse. Thus, mucus barrier leakage may indicate a clinical relapse of CD. In addition, the pH and ion concentration of mucus is reportedly associated with the penetration of microbes [82].

Previous research has indicated mucin glycosylation importance for maintaining mucin’s normal functioning [83, 84]. This process protect the mucin’s protein core from degradation by proteases produced by the microbiota [85]. An abnormal status of mucin glycosylation is associated with increased inflammation and enhanced epithelial permeability in patients with IBD [84, 86]. Additionally, there are differences in the mucin composition between CD patients and healthy individuals. CD patients exhibit abnormal expression of genes such as MUC1 mRNA, MUC2, MUC3, MUC5AC, and MUC5B [86–88]. Besides, the glycan on mucin may also be altered during CD. The O-linked glycan is the main group changed during the disease process [89]. Previous research reported that the sulfate groups on mucin glycans are reduced. Meanwhile, the ratio of sialomucin to sulfomucin is elevated, which might disturb the negative charges of the mucin layer [90, 91]. The fucose and galactose of glycans on mucin are also decreased [92]. All these alterations consequently lead to the protection capacity of mucin being weakened. Further investigation is needed to ascertain how these differences in mucin composition contribute to the pathogenesis of CD.

Inflammatory cytokines, including TNFα, interferon (IFN)-γ, and IL-6 upregulate MUC2 and MUC3 synthesis and secretion [93, 94]. Therefore, in patients with CD, the mucin layer shows destruction with abnormal glycosylation of mucin [90, 95–97]. It has been indicated that the primary impact is on the O-glycosylation of MUC2 [84]. This, in turn, can result in an impaired function of mucin within the mucus layer. In contrast, upregulated mucin synthesis and secretion by goblet cells may exaggerate protein misfolding, leading to CD. The mucin TFF3 induces cell migration and inhibits cell apoptosis [98], which worsens the cleavage efficiency of misfolded proteins. However, TFF3 may also positively impact disease etiologic factors by promoting mucosal injury healing and eliminating bacterial toxins [99]. This was supported by a study with intestinal TFF3-deficient mice, which observed poor mucosal healing following epithelial damage due to dextran sulfate sodium (DSS) intake [100]. Hence, a consensus has not been reached regarding whether mucus is the cause or result of CD. Moreover, the effects of different types of mucins on CD have not been elucidated. Thus, further studies are required to determine the relationship between mucins and CD.

### Goblet cells dysfunction could contribute to the irregular immune response, which finally lead to the CD

An aggressive acquired immune response is a vital factor related to CD pathogenesis [7]. Mucin and mucus secreted by goblet cells are associated with the regulation of exaggerated immune responses, which is a common phenomenon in CD. Bergstorm evaluated the relationship between goblet cells and CD4^+^ immune cell recruitment in inflammatory bowel disease [101]. Resistin-like molecule beta (RELEM-β), a kind of mucin secreted by goblet cells, was significantly induced during *Citrobacter rodentium* infection. Moreover, in RELEM-β gene deficiency mice, CD4^+^ immune cell recruitment was decreased, leading to a decrease in IL-22 and intestinal epithelial cell proliferation. These findings further support the notion that goblet cells and the substances they secrete are related to immune responses.

In addition to the CD4^+^ recruitment pathway, goblet cells may also affect the immune response in CD through dendritic cells. Goblet cells serve as antigen-presenting cells that transport the antigen to CD103^+^ DCs in the gut. These CD103^+^ DCs then facilitate the differentiation of FOXP3^+^ regulatory T cells by utilizing a pathway dependent on TGF-β and retinoic acid [102–104].By sensing and passing luminal antigens to CD103^+^ dendritic cells [33, 35], goblet cells help the immune system mature in the gut. The immune system develops and is maintained at normal levels through antigen presentation. As a result, immune tolerance is established. Goblet cell dysfunction disrupts this process, leading to an aggressive immune response against commensal bacteria. Consequently, patients with abnormal goblet cell functions may experience a breakdown in immune tolerance, particularly in the case of CD. Another indirect influence of goblet cells on the pathogenic immune response is mediated by the downstream signaling pathway of dendritic cells. As previously mentioned, dendritic cells promote the development of Foxp 3 + T regulatory cells (Tregs) with the help of transforming growth factor (TGF)-β, dietary metabolites, and retinoic acid [102, 104]. Meanwhile, α4β7 is downregulated in Tregs of CD patients, which may lead to limited migration of T_reg_s to the inflammatory sites [105]. Therefore, abnormal goblet cell-associated antigen presentation may indirectly lead to CD through dendritic cells.

Goblet cells may also indirectly affect CD pathogenesis through cytokine and dendritic cell-related T-cell pathways. Previous studies have shown that goblet cells can induce several cytokines, which contribute to the regulation of immune responses [106]. Cytokines are closely associated with CD pathogenesis. IL-12 levels are higher in CD patients than in the control group [107–109], whereas anti-IL-12 treatment relieves CD inflammation [110]. Indeed, cytokines are the driving factors of T helper cells in CD [111]. Since CD is a type of Th1 cytokine-mediated disease [112], abnormal cytokine secretion by goblet cells indirectly contributes to the overreactive inflammatory response.

### ER stress and autophagy during goblet cells synthesis process is closely linked with CD

Endoplasmic reticulum (ER) stress and autophagy in goblet cells may contribute to CD onset. Heazlewood analyzed the endoplasmic constitution of missense MUC2-mutated mice [113]. In these mice, the MUC2 precursor accumulated in the endoplasm of goblet cells, and biochemical evidence of endoplasmic stress was observed, leading to the spontaneous development of colitis. Endoplasmic stress and MUC2 misfolding are thought to induce IBD in mice. However, it is uncertain whether these processes lead to pathogenesis in humans. Meanwhile, the deletion of the transcription factor XBP1 elucidated a close relationship between ER stress during the synthesis process and CD in humans [114]. In particular, ER stress in goblet cells contributes to CD development. These misfolded mucins accumulate in the ER and are transported via Sect. 61 translocation. Misfolded mucins are lysed via autophagy or ubiquitination in the cytoplasm.

Autophagy in goblet cells can be regarded as a protective process to prevent inflammation, which ultimately prevents CD. A study revealed that autophagy, ER stress, and IBD are closely associated [115]. It was found that the escalation of autophagy plays a crucial role in alleviating ER stress [76]. Stimulates mucus secretion when working with the CD risk gene NOD2. Consequently, sufficient mucus secretion serves as a protective mechanism against inflammation in the gut. The disease process of IBD is improved after TREM-1 inhibition, and autophagy is subsequently restored. Meanwhile, Sun and colleagues claimed that a two-way regulation exists between inflammasomes and autophagy [116]. Inflammasomes can become decreased through autophagy by removing endogenous damage-associated molecular patterns from mitochondria. However, inflammasomes can also regulate the autophagy process directly or indirectly through the ubiquitin sensor p62 or caspase-1 pathway. Moreover, excessive autophagy may cause mucus barrier damage, leading to CD pathogenesis instead. Thus, it is vital to maintain autophagy in goblet cells within the normal range to prevent CD.

### Goblet cells abnormal interactions with microbiome leading to CD

The microbiome is a crucial factor in CD pathogenesis. Increased numbers of mucosal-associated invasive and adhesive bacteria have been discovered in CD [117, 118]. For instance, Dirk Gevers collected and analyzed samples from different locations in the gastrointestinal tract before treatment and found that *Pasteurellaceae*, *Enterobacteriaceae*, *Fusobacteriaceae*, and *Veillonellaceae* were predominant, whereas the abundance of *Bacteroidales*, *Erysipelotrichales*, and *Clostridiales* decreased. Hence, the abundance of commensal bacteria decreased, while that of pathogenic bacteria increased in CD, even in the early stages [119]. Goblet cells and the mucus they secrete are crucial in maintaining the mucus barrier and selecting the bacteria that colonize the intestinal tract. Dysbiosis can further result in abnormal immune development. Consequently, dysfunctional goblet cells interacting with the microbiome can lead to dysbiosis, disruption of the mucus barrier, and dysregulation of the immune system.

When goblet cells are dysfunctional, it can lead to gut dysbiosis, compromising the integrity of the mucus layer and making it more susceptible to pathogenic bacteria. Consequently, this creates a vicious cycle that exacerbates the pathogenesis of CD. One specific bacterial species of note is adherent-invasive *Escherichia coli* (AIEC), which is more abundant in CD patients [120, 121]. The increased adherence and invasion capabilities of AIEC enable these bacteria to penetrate the mucus layer more easily [122].

Another study conducted highlighted that compared to healthy individuals, patients with CD and their relatives exhibit enhanced mucin degradation capacity in their gut bacteria, facilitating the breakdown and penetration of the protective mucus barrier [123]. Moreover, bacteria are recognized by two receptors, nucleotide-binding oligomerization domain-containing protein 2 (NOD2) and Toll-like receptors (TLRs). These receptors promote mucus secretion by goblet cells. However, patients with Crohn’s disease exhibit mutations in NOD2/CARD15 and TLRs [124, 125]. The invasion of bacteria triggers the innate immune response, causing chronic inflammation in these patients. Apart from receptor-related pathways for pathogen-associated molecular patterns, specific sulfate-reducing bacteria convert sulfate to sulfide, breaking the disulfide bond within the mucus and leading to an abnormal mucus structure, thereby contributing to the development of Crohn’s disease [126]. Consequently, dysfunction in goblet cells can disrupt the mucus layer, resulting in bacterial invasion and fostering the progression of Crohn’s disease.

In addition to the dysfunction of goblet cells and dysbiosis-induced mucus penetration, another potential mechanism contributing to the pathogenesis of CD is dysbiosis-associated aberrant immune system development. While virulent pathogenic bacteria can trigger intestinal inflammation, it is important to note that the microbiome plays a critical role in the normal development of the human immune system, particularly within the intestines [127]. It was revealed that the colonization of the microbiota is essential for inducing a tolerogenic phenotype in immune cells within the intestinal tract [128]. However, dysbiosis disrupts the normal gut immune system development, leading to an exaggerated immune response against commensal bacteria and ultimately resulting in CD.

## Treatment for Crohn’s disease related to goblet cells

Currently, many drugs and food therapies involving goblet cells have been adopted for the treatment of CD (Table 1). These drug therapies may have mechanisms related to the goblet cell signaling pathway or function in CD and potential drug targets have been discovered. The mechanisms and routes these therapies take for restoring goblet cell’s function were classified as “Direct” or “Indirect” in the last column of Table 1. The therapy targeting at goblet cells and directly promoting goblet cell’s function or helping the dysfunctional goblet cell regaining its function will be labeled “Direct”. For the therapy aimed at eliminating the upstream factor (e.g. inflammation) which led to the dysfunctional goblet cell and thus improving goblet cells related functions are labeled “Indirect”.

**Table 1:** Treatments for Crohn’s Disease related to Goblet cells

Name	Effects	Direct or indirect influence on goblet cells
**Current pharmacotherapies**		
Infliximab Ustekinumab Risankizumab	Through the process of anti-IL-12 and IL-23, goblet cells proliferation and mucus healing were facilitated	Indirect
Tofacitinib Filgotinib	Through inhibiting JAK, mucosal healing was promoted	Direct
**Potential substances and targets**		
Probiotics	Commensal bacteria repopulation	Indirect
Antibiotics	Pathogenic bacterium elimination might be helpful for the treatment. But it may also lead to dysbiosis. Further study should be made in the future	Indirect
Butyrate	Induce macrophages polarization, facilitate cell proliferation and mucus secretion	Indirect
PPARγagonist	Promote mucus secretion	Direct
Farnesoid X receptor agonist	Less goblet cells loss and less inflammatory cells infiltration	Unclear
Nuclear factor-KappaB	Recover goblet cells function and proliferation	Indirect
Nitrate	Increase mucus layer thickness and keep goblet cells abundant	Unclear
IL-13 receptorα2 blocker	Restore the mucus layer more quickly	Direct
Thymopentin	By inducing IL-22 production, it stimulates the secretion of mucus	Indirect
IL-10	Suppress endoplasmic reticulum stress	Direct
Glutamine		
Glucocorticoids		
Estrogen		
4-phenylburyrate		
Tauroursodeoxycholic acid		
**Dietary and nutrient therapy**		
Arctium lappa L	Promote mucus secretion and increase goblet cells number	Direct
3-glucoside-enriched strawberry	Inhibit proinflammatory cytokines and promote mucus secretion	Indirect
Dietary grape seed extract	Increase goblet cells number and decrease the expression of claudin 2 mRNA	Indirect
Lentinula edodes extract	Increase goblet cells number and reduce infiltration of inflammatory cells	Indirect
Royal jelly	Detailed mechanism remained unclear.	Unclear
Dietary antioxidant micronutrients		Unclear

### Current pharmacotherapies

Several drugs are in use within clinical settings to treat CD, and the mechanism of some may be partially associated with goblet cells. Infliximab, Ustekinumab, and Risankizumab belong to the anti-IL-12/23 drug category. Previous research has indicated that these drugs act by blocking cytokines in combination with epithelial cells [129–132]. This pathway enhances mucosal healing, which may be related to the secretion of goblet cell mucus. Additionally, JAK inhibitors, such as Tofacitinib and Filgotinib, which inhibit the downstream signaling pathway, promote mucosal healing [133–135].

### Potential substances and targets

During commensal bacteria dysbiosis, the pathobionts could release virulence factor, thus aggravating CD severity [136]. Moreover, previous research indicated that the resident H2S producing bacteria, such as C.innocuum, could indicate CD severity [137]. Thus, CD severity is correlated with the extent of dysbiosis to some extent. According to research by Boyapati R et al., commensal bacteria repopulating methods like probiotics or fecal microbiota transplantation have been considered potential treatments for CD [138]. This point was verified by another study, which indicated that MUC2 and MUC3 expression could be induced after using probiotics Lactobacillus plantarum [139]. Thus, the probiotics could improve CD symptom via altering mucin expression. Moreover, Boyapati R et al. also concluded that pathogenic bacteria elimination methods like antibiotic treatment aimed at AIEC would be helpful for CD treatment. However, another research indicated that antibiotics may exaggerate the dysbiosis associated with CD [119]. Since there is no consensus on the use of antibiotics, further studies should be conducted to verify this point in the future.

**Fig. 3:**
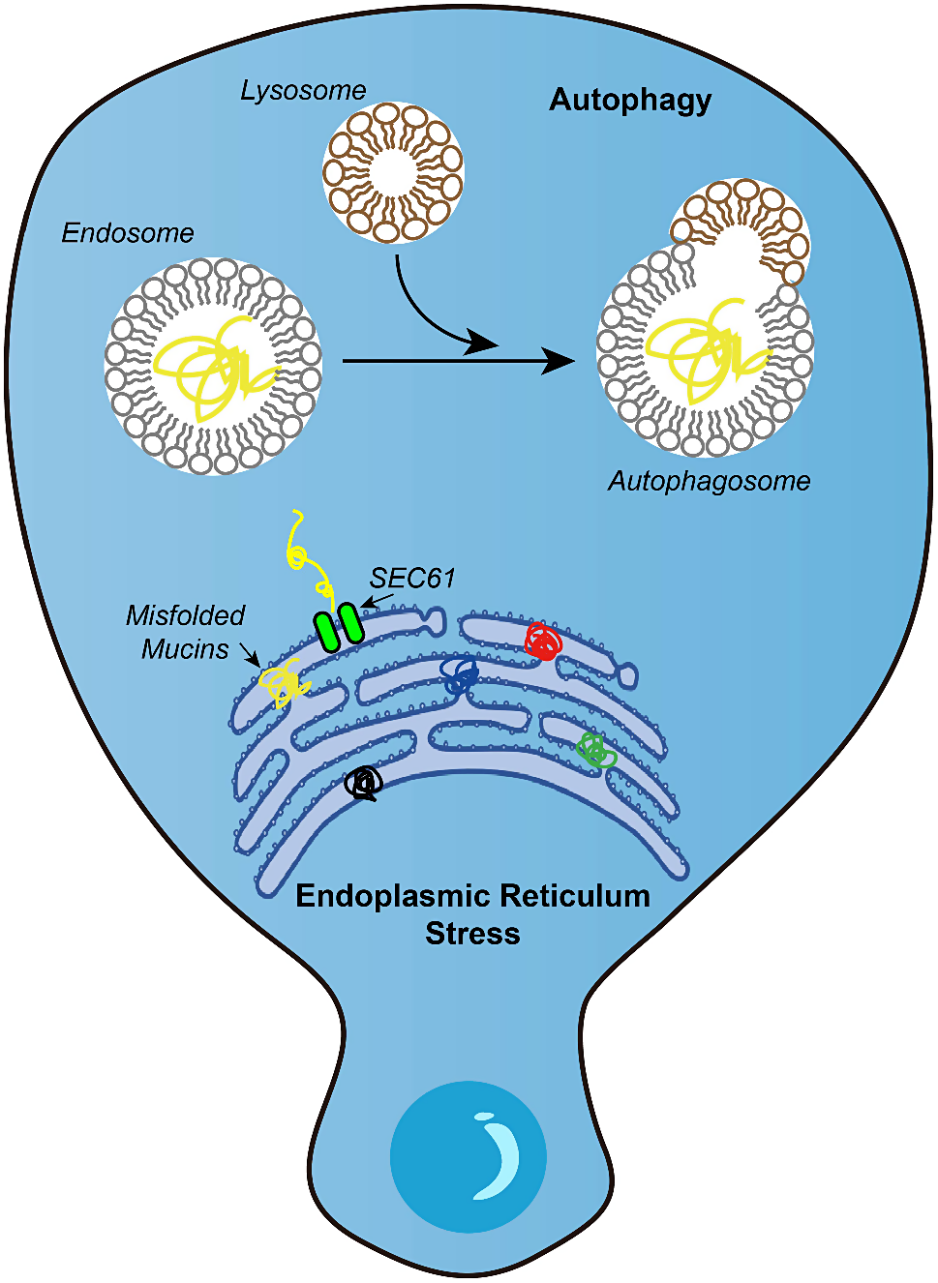
ER stress and autophagy in the goblet cells. Mucin misfolding in the endoplasmic reticulum will lead to the ER stress in goblet cells.ER stress will further lead to inflammatory bowel disease including Crohn’s disease. Autophagy, a cell biological process is a solution to this situation. During autophagy, endosome wrap misfolded mucin up and fuse with lysosome, which formed autophagosome. After the lysis process, the misfolded mucin was vanished in the autophagosome

Liping et al. pointed out that butyrate derived from gut microbiota may increase the mucus production and cell proliferation of goblet cells [140]. In this process, the butyrate induces the polarization of M2 macrophages, which further facilitates goblet cell proliferation and mucus secretion in DSS-induced colitis models by activating the WNT-ERK1/2 pathway. Butyrate may have a therapeutic effect on CD by improving the function and proliferation of goblet cells (Fig. 3).

PPARγ has been regarded as a promising target for CD treatment [141]. In a previous study, NLRP6 was shown to play a vital role in goblet cell secretion of mucus and autophagy. Ranson and colleagues discovered that PPARγ agonist binds to the promotion site of NLRP6 [142]. NLRP6 in sentinel goblet cells will promote the exocytosis of the mucus upon sensing TLR ligands [143]. Meanwhile, PPARγ expression is suppressed in CD patients. Thus, PPARγ serves as a mucus barrier coordinator.

The farnesoid X receptor (Fxr) is another novel target for the pharmacological treatment of goblet cells. Gadaleta and colleagues focused on the effects of Fxr agonists [144]. The mice included in the study were divided into wild-type and Fxr-null groups, treated with DSS and trinitrobenzene sulfonic acid (TNBS), respectively, to generate a colitis model. All subjects were treated with the Fxr agonist INT747 or vehicle. INT747-treated wild-type mice exhibited less goblet cell loss, less inflammatory cell infiltration, and lower mucus barrier permeability. Therefore, Fxr agonists may act on goblet cells and relieve CD pathogenesis through various pathways. However, the mechanisms underlying these pathways remain unclear and require further investigation.

Transcriptional factor nuclear factor-kappa B (NF-κB) ameliorates IBD [145]. In DSS and TNBS-induced colitis models, an NF-κB decoy helps to restore colonic homeostasis. Moreover, treatment with an NF-κB decoy restored the function of goblet cells in mice and limited the inflammatory reaction. This may be achieved by NF-κB regulating the TNFα, IL-6, and IL-1β inflammatory cascade. The expression of intestinal trefoil factor (ITF) was also found to increase. Hence, NF-κB might promote mucosal epithelium cell proliferation through ITF. Accordingly, De Vry et al. concluded that an NF-κB decoy may be taken as a cross-functional therapeutic for IBD.

Nitrate supplementation is another potential method for CD remission. In a previous study [146], DSS-induced colitis mice were treated with nitrate or nitrite in their drinking water for a week. Nitrate supplementation was found to prevent a decrease in the mucus layer and maintained goblet cell abundance among intestinal goblet cells in the colitis model. Therefore, nitrate may exert therapeutic and preventive effects on CD by influencing goblet cells. This mechanism may be related to the inhibitory effects of nitric oxide (NO)-containing compounds on colonic inflammation. However, the exact route of nitrate uptake via the NO-mediated pathway remains unclear.

Mucosal IL-13 receptor alpha 2 (IL-13Rα2) expression has previously been used as a predictive marker for infliximab therapy in unresponsive individuals. Further research was conducted by Verstockt and colleagues to figure out the relationship between IL-13Rα2 and IBD pathology [147]. Wild-type and IL-13Rα2 knock-out DSS-induced colitis mice were compared and showed IL-13Rα2 knock-out group recovered more rapidly from colitis. Goblet cells and mucus were also recovered more rapidly in the IL-13Rα2 knock-out group than wild type group. A negative correlation between mucosal IL-13Rα2 mRNA and mRNA of mucus barrier, goblet cell-specific genes and goblet cell number was revealed. These results indicated that IL-13Rα2, a receptor related to goblet cell normal function and mucus barrier recovery, is a potential target whose blockage may be effective for the treatment of Crohn’s disease.

The mucosal healing process is essential in the treatment of Crohn’s disease. Previous research based on organoids and resections of the human intestine and mice revealed that IL-22 promotes mucus secretion and regeneration of the intestinal epithelial layer in CD and ulcerative colitis [148]. It is investigated the effectiveness of thymopentin in the treatment of ulcerative colitis and found that thymopentin prevents DSS-induced colitis in mice by inducing the production of IL-22 [149]. As IL-22 can also relieve CD, thymopentin may be a potential drug therapy for CD. However, further experiments are required to verify the effectiveness of this approach.

As mentioned above, ER stress in goblet cells is a crucial factor leading to CD pathogenesis. Meanwhile, IL-10 reportedly has a suppressive effect on ER stress [150]. By analyzing and comparing the effects of the IL-10 signaling pathway in Winnie and wild-type mice, it was shown that IL-10 maintained the folding and transportation of MUC2 in the ER of goblet cells. Under ER stress, the IL-10R1-mediated signaling pathway activates various transcription factors, including signal transducer and activator of transcription 1 (STAT1) and STAT3. Subsequently, the synthesis of proteins that promote correct folding, such as anterior gradient protein 2 (AGR2), is increased. The ER-associated degradation of misfolded proteins is also enhanced. Thus, the IL-10 signaling pathway may be a potential therapeutic target for CD treatment. Additionally, glutamine, glucocorticoids, estrogen, 4-phenylbutyrate, and tauroursodeoxycholic acid have all been shown to alleviate ER stress [150–154]. A small molecule drug targeting unfolded protein response sensors and inhibiting eIF2alpha dephosphorylation has also been reported as effective for promoting cell survival during ER stress [155]. These substances, which target goblet cells, could serve as potent therapies for Crohn’s disease by addressing ER stress.

Although cytokine therapy is an effective treatment for CD, maintaining stability in vivo and during delivery is worthy of consideration. To address these issues, Hamady and colleagues tested an engineered commensal gut bacterium for cytokine delivery [157]. They found that the engineered recombinants accelerated epithelial healing. Thus, this commensal bacterial engineering method might prove effective for the stable delivery of cytokines.

### Dietary and nutrition therapy

Food therapy is useful for the treatment of CD [158]. Several food therapy methods involving goblet cells have been developed. For instance, *Arctium lappa L*.- a vegetable rich in the phytoestrogen arctigenin-has great health benefits [159]. Tao and colleagues evaluated the therapeutic effects of phytoestrogens in IBD and found that, by inhibiting goblet cell apoptosis, arctigenin promotes mucus secretion and increases the abundance of goblet cells [160]. The mucus barrier is preserved via the Erβ/tripartite motif containing-21 (TRIM21)/prohibitin 1 (PHB1) pathway. Hence, arctigenin may represent a potential drug for the treatment of CD.

Pelargonidin 3-glucoside-enriched strawberries inhibit pro-inflammatory cytokines, including TNF and cyclooxygenase 2 (COX2), and may also promote mucus secretion via IL-10-mediated pathways [161]. A dietary grape seed extract has also been shown to increase the number of goblet cells and decrease claudin 2 mRNA. Claudin functions in tight junction weakening, which increases the mucus barrier permeability [162]. Another study indicated that lentinula edodes extract reduces the infiltration of inflammatory cells and increases the number of goblet cells [163]. Meanwhile, other types of food, such as royal jelly [164] and dietary antioxidant micronutrients [165], exhibit positive effects on goblet cell functioning. In contrast, a high-fat diet induces ER stress via free fatty acids in goblet cells and alters the microbiome constitution [166]. Consequently, ER stress and the altered microbiome led to inflammation, which worsens CD.

## Conclusions

Here, we reviewed the function of goblet cells and their relationship with CD. Goblet cells play a vital role in maintaining the stability of the gut microbiome, while abnormal goblet cell function leads to dysbiosis. However, certain bacteria have a negative effect on mucus and goblet cells, leading to a bacteria-related CD etiology. Goblet cells interact with the immune system to facilitate antigen presentation and cytokine-mediated inflammatory responses. Dysfunction of both pathways leads to CD due to T_reg_ migration failure, an anti-commensal bacterial response, and an abnormal cytokine mediated Th1 immune response. These processes contribute to the immune-associated etiology of CD. Goblet cells also play a key role in regenerating the mucosal barrier and secreting mucin to protect enterocytes from invading pathogenic bacteria. However, misfolded proteins during mucin synthesis cause ER stress, which contributes to CD develop pment. Hence, many therapeutic modalities targeting sites have been found to be related to these goblet cell-related CD etiological factors. However, further investigation is needed to elucidate the detailed mechanisms underlying the effects of goblet cells on CD. Collectively, this review will inform the development of effective goblet cell-targeting therapeutics for the clinical treatment of CD.

## Data Availability

Not applicable.
